# Physical Training Chronically Stimulates the Motor Neuron Cell Nucleus in the Ts65Dn Mouse, a Model of Down Syndrome

**DOI:** 10.3390/cells12111488

**Published:** 2023-05-27

**Authors:** Chiara Rita Inguscio, Maria Assunta Lacavalla, Barbara Cisterna, Carlo Zancanaro, Manuela Malatesta

**Affiliations:** Department of Neurosciences, Biomedicine and Movement Sciences, Anatomy and Histology Section, University of Verona, Strada Le Grazie 8, I-37134 Verona, Italy; chiararita.inguscio@univr.it (C.R.I.); mariaassunta.lacavalla@univr.it (M.A.L.); barbara.cisterna@univr.it (B.C.); manuela.malatesta@univr.it (M.M.)

**Keywords:** cell nucleus, Down syndrome, motor neuron, physical exercise, transmission electron microscopy, Ts65Dn mouse

## Abstract

Down syndrome (DS) is a genetically-based disease based on the trisomy of chromosome 21 (Hsa21). DS is characterized by intellectual disability in association with several pathological traits among which early aging and altered motor coordination are prominent. Physical training or passive exercise were found to be useful in counteracting motor impairment in DS subjects. In this study we used the Ts65Dn mouse, a widely accepted animal model of DS, to investigate the ultrastructural architecture of the medullary motor neuron cell nucleus taken as marker of the cell functional state. Using transmission electron microscopy, ultrastructural morphometry, and immunocytochemistry we carried out a detailed investigation of possible trisomy-related alteration(s) of nuclear constituents, which are known to vary their amount and distribution as a function of nuclear activity, as well as the effect of adapted physical training upon them. Results demonstrated that trisomy per se affects nuclear constituents to a limited extent; however, adapted physical training is able to chronically stimulate pre-mRNA transcription and processing activity in motor neuron nuclei of trisomic mice, although to a lesser extent than in their euploid mates. These findings are a step towards understanding the mechanisms underlying the positive effect of physical activity in DS.

## 1. Introduction

Down syndrome (DS) or trisomy of chromosome 21 (Hsa21) is a genetically-based disease affecting about 1 in 700 human newborns [1]. Normal gene expression is altered in DS, which results in intellectual disability and several pathological traits, including craniofacial alterations and congenital heart disease, early onset Alzheimer’s disease, and gastrointestinal disorders. In addition, individuals with DS have (although with high interindividual variability) altered motor coordination [2,3], the most characteristic feature being slowed voluntary movement. Accordingly, persons with DS need longer times to react to a stimulus, and even longer to accomplish a motor task. Moreover, persons with DS present muscle hypotonia, low muscle strength, and ligament laxity [4,5], which all contribute to compromise their gait patterns [6]. Further, persons with DS exhibit premature aging at multiple organ levels [7,8]; this inevitably also affects muscle mass, strength, and function, similarly to the physiologically occurring sarcopenia in healthy aging subjects [9] and further contributes to motor impairment in DS.

Physical training or passive exercise were found to be useful in counteracting muscle hypotonia in DS babies [10,11], as well as mitigating motor disabilities and increasing muscle strength in DS adolescents [12,13], and improving several outcomes in persons with DS [14]. Physical exercise proved to be beneficial to muscle mass and function in sarcopenia of aging, (recent review in [15]), as well as motor coordination and learning [16]. Due to obvious ethical problems, these kinds of studies only provide very limited morphological evidence for the effects of physical exercise on either skeletal muscle, or the areas of the central nervous system (CNS) involved in motor function. Instead, animal models are suitable tools to this aim. 

The Ts65Dn mouse (bearing a trisomy for a segment of chromosome 16 that contains genes orthologous to Hsa21) [17] is a widely accepted model of DS since it is fairly reminiscent of the human DS phenotype [18], including gross motor and muscle features [19,20], as well as deficit in grip strength, motor coordination, and running and swimming speeds [21]. Ts65Dn mice also show significant delay in the development of a number of sensory and motor tasks [19,22]. Ts65Dn mice were found to show structural and compositional alterations of the skeletal muscles [23] of reminiscent age-related sarcopenia [24]. Some studies demonstrated neurological benefits after physical exercise in the Ts65Dn mouse [25,26,27]. Adapted physical training, which is better accepted by the Ts65Dn mouse, also demonstrated some beneficial effects on hindlimb muscles investigated with nuclear magnetic resonance [28] or transmission electron microscopy [29].

To deepen our knowledge on the structural basis of neuromuscular deficits in DS and the potential benefit of physical training, we moved our attention from muscle to medullary motor neurons. In the present work, we focused on the ultrastructural architecture of the medullary motoneuron cell nucleus taken as marker of the cell functional state. Intriguingly, a relationship between nuclear structural and functional alterations in hippocampal neurons, and cognitive dysfunctions were recently suggested in trisomic Ts65Dn mice [30]. Using transmission electron microscopy, ultrastructural morphometry, and immunocytochemistry we carried out a detailed investigation of possible trisomy-related alterations of nuclear constituents by analysing heterochromatin clumps, nucleoplasmic RNP-containing structures (perichromatin fibrils, perichromatin granules, and interchromatin granules), as well as nucleolar structural components (fibrillar centres; dense fibrillar component; granular component, see [31] for this nomenclature), which vary their amount and distribution according to the nuclear activity (e.g., [32,33,34,35]). The effect of adapted physical training on the above nuclear constituents was evaluated as well.

## 2. Materials and Methods

### 2.1. Animals and Physical Activity 

Ts65Dn [strain: B6EiC3Sn.BLia-Ts(17<16>)65Dn/DnJ] breeder mice were obtained from the Jackson Laboratory, ME, USA. The colony was maintained by breeding trisomic female mice to euploid B6EiC3Sn.BLiAF1/J males. Pups were weaned at 21 days of age. Tissue for genotyping was obtained from tail clips in p11 mice. Genotyping was accomplished by Mmu17 translocation breakpoint separated PCR [36]. Eight (four trisomic and four euploid) male Ts65Dn mice aged 8 ± 3.10 months were used in this work. They were housed by genotype under standard conditions (24 ± 1 °C ambient temperature, 60 ± 15% relative humidity, and 12 h light/dark cycle) and fed ad libitum with standard commercial chow. The trisomic mice presented deficits in balance and motor coordination by 4 months of age accordingly with previous studies [19]. Four animals (two euploid and two trisomic, hereinafter referred to as sedentary euploid and sedentary trisomic, respectively) had only spontaneous free-moving activity in the cage, while other four animals (two euploid and two trisomic, hereinafter referred to as trained euploid and trained trisomic, respectively) run on a Harvard Instruments treadmill (Crisel Instruments, Rome, Italy) 45 min a day at 8 m × min^−1^ belt speed (0% incline), 5 days a week for 1 month [28]. Current treadmill protocols for adult mice consistently use 1 h running a day at belt speed > 10 m × min^−1^. In this work, the physical training was adapted to optimize trisomic mice compliance to training. To avoid the confounding acute effect of running vs. the chronic effect of physical training, all animals were analysed three days after the last treadmill training session.

The experimental protocol was approved by the Italian Ministry of Health (ref.: 538/2015-PR). 

### 2.2. Tissue Processing

Ts65Dn mice were deeply anesthetized using Tribromoethanol drug and perfused transcardially with 0.1 M phosphate-buffered solution (PBS) followed by 4% paraformaldehyde in PBS. After perfusion, the spinal cord was quickly and gently removed, and the distal half was transversally cut into about 2 mm long segments. Samples were then placed for 2 h at 4 °C in either a 2.5% glutaraldehyde plus 2% paraformaldehyde solution (samples intended for ultrastructural morphology and morphometry) or 4% paraformaldehyde and 0.2% glutaraldehyde in 0.1M PBS (samples intended for ultrastructural immunocytochemistry). After fixation, samples for ultrastructural morphology were rinsed with PBS, postfixed with 1% OsO4 for 2 h at 4 °C, dehydrated with graded acetones and embedded in Epon 812 resin. For immunohistochemistry, samples were washed in PBS, treated with 0.5 M NH_4_Cl solution in PBS for 45 min at 4 °C to block free aldehyde groups, dehydrated in graded concentrations of ethanol at room temperature, and embedded in London White resin (LRW). The samples were then cut into 2 µm thick sections stained with 1% aqueous toluidine blue to identify motor neurons in the anterior horns of grey matter and properly trim the resin bloc. Then, for ultrastructural morphology, ultrathin (70–90 nm thick) sections of Epon-embedded samples were stained with Reinhold’s lead citrate for 1 min and observed in a Philips Morgagni transmission electron microscope operating at 80 kV and equipped with a Megaview III camera for digital image acquisition. For immunocytochemistry, ultrathin sections of LRW embedded tissue were processed as described below.

### 2.3. Ultrastructural Morphometry

Morphometric analysis was conducted on transmission electron micrographs (×18,000) by using Image J image analysis software (NIH). A total of 80 motor neuron nuclei were analysed (10 nuclei per animal). Area and perimeter of nuclei were measured, and the index of nuclear shape irregularity was expressed as the ratio between the perimeter and the circumference of the equivalent circle (I = P/2πr, where P is the measured perimeter and r is the radius of the equivalent circle having the same area). The area of nucleoli and their structural components, i.e., fibrillar centres, dense fibrillar component and granular component were also measured and the percentage of fibrillar centres, dense fibrillar component and granular component area per nucleolus was calculated. The nuclear pores were counted and their frequency was expressed as the ratio between pore number and the nuclear membrane length (nuclear pores/µm). Moreover, the area of condensed chromatin clumps was measured and expressed as the percentage of nuclear area occupied by condensed chromatin as well as used to calculate the nucleoplasmic area (nuclear region devoid of heterochromatin and nucleolus). The area of each interchromatin granule cluster was also measured and summed up to calculate the percentage of nucleoplasmic area occupied by interchromatin granules. Finally, perichromatin granules were counted and their density expressed as the ratio between perichromatin granules number and the nucleoplasmic area (perichromatin granules/µm^2^).

### 2.4. Ultrastructural Immunocytochemistry

Ultrathin sections were processed for immunocytochemistry by using mouse monoclonal antibodies directed against phosphorylated polymerase II (Abcam, Cambridge, MA, USA; ab24759), the activated form of the enzyme occurring at pre-mRNA transcription sites [37,38], (Sm)snRNP (small nuclear RiboNucleoProtein) core protein (Abcam, ab3138), involved in the early splicing of pre-mRNA [39] or rabbit polyclonal antibody directed against fibrillarin (Cytoskeleton Inc., Denver, CO, USA), an early splicing factor of pre-rRNA [40]. Sections were floated for 3 min on normal goat serum (NGS) diluted 1:100 in PBS and then incubated for 17 h at 4 °C with the primary antibodies, all diluted 1:10 in PBS containing 0.1% bovine serum albumin (Fluka, Buchs, Switzerland) and 0.05% Tween 20. Then, sections were rinsed with PBS, floated for 3 min on NGS, and reacted for 20 min at room temperature with the secondary 12 or 6 nm gold-conjugated antibody (Jackson ImmunoResearch Inc., West Grove, PA, USA) diluted 1:20 in PBS. Finally, sections were rinsed in PBS and distilled water and air dried. As the control, some grids were treated with the incubation mixture without the primary antibody and then processed as described above. To clearly identify the nuclear structural constituents containing RNPs, all immunolabeled sections were stained with Uranyl Acetate Replacement Stain (Electron Microscopy Sciences, Hatfield, PA, USA) and lead citrate according to [41]. The specimens were observed in the Philips Morgagni transmission electron microscope.

To quantify immunolabelling, colloidal gold particles density was evaluated on sections from sedentary euploid, sedentary trisomic, trained euploid, and trained trisomic mice treated in the same run. Immunolabelling for polymerase II and (Sm)snRNP core protein was evaluated over the interchromatin space (i.e., the nucleoplasmic region devoid of heterochromatin clumps) and that for fibrillarin over the nucleolar area. For each antibody, areas were measured on 20 randomly selected electron micrographs (22,000×) from sedentary euploid, sedentary trisomic, trained euploid, and trained trisomic mice using a computerized image analysis system (AnalySIS Image processing, Soft Imaging System GmbH, Münster, Germany). For background evaluation, the resin outside the tissue was considered. The gold particles present over the investigated compartment were counted, and the labelling density was expressed as the number of gold particles/µm^2^. 

### 2.5. Statistics

The data for each variable were pooled according to the experimental animal group (sedentary euploid, sedentary trisomic, trained euploid, and trained trisomic), and the mean ± standard error of the mean (SEM) values calculated. The Shapiro–Wilk test showed that data for all measured variables were not normally distributed (*p* < 0.001). Consequently, statistical analysis was performed using the non-parametric Kruskal–Wallis test. Where necessary, post hoc group–group comparisons was carried out using the Mann–Whitney test. Significance was set at *p* ≤ 0.05.

## 3. Results

### 3.1. Ultrastructural Morphology

**Figure 1:**
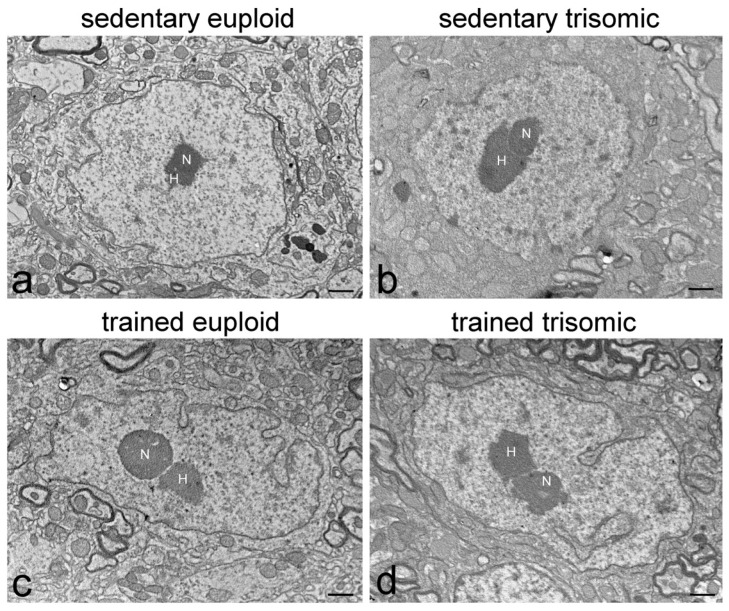
Transmission electron micrographs of motor neuron nuclei of sedentary euploid (**a**), sedentary trisomic (**b**), trained euploid (**c**), and trained trisomic (**d**) mice. Note the irregular nuclear shape in trained animals (**c**,**d**). N, nucleolus; H, heterochromatin clumps. Bars, 1 µm.

**Figure 2:**
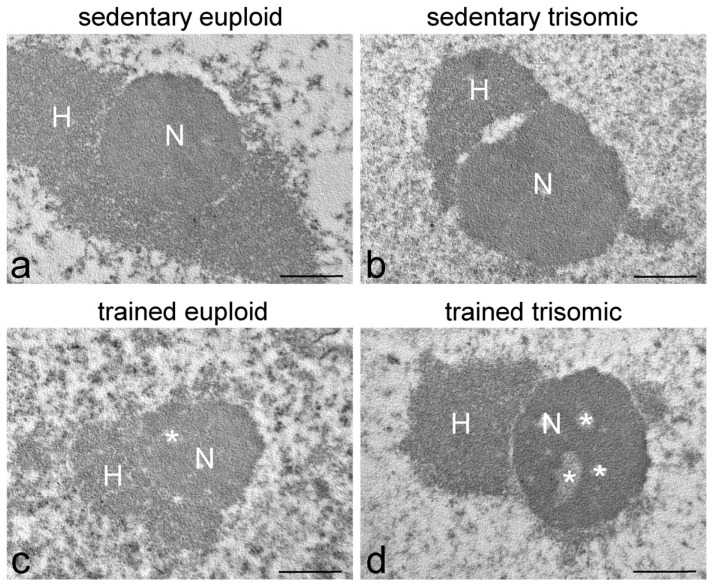
Transmission electron micrographs of motor neuron nucleoli of sedentary euploid (**a**), sedentary trisomic (**b**), trained euploid (**c**), and trained trisomic (**d**) mice. Asterisks indicate fibrillar centre. N, nucleolus; H, heterochromatin clumps. Bars, 500 nm.

In sedentary euploid mice, motor neuron nuclei were characterised by roundish shape and slightly irregular border (Figure 1a). Heterochromatin was distributed in small clumps both at the nuclear periphery and associated with the nucleolus. In the nucleoplasm, clusters of interchromatin granules were evident, perichromatin granules were numerous and preferentially distributed at the edge of heterochromatin clumps, while perichromatin fibrils were quite scarce. Generally, the nuclei contained one single, centrally located roundish and compact nucleolus (Figure 1a and Figure 2a). The nucleoli contained few small fibrillar centres surrounded by dense fibrillar component, while granular component was prominent.

Motor neuron nuclei in sedentary trisomic and sedentary euploid mice (Figure 1a,b and Figure 2a,b) showed similar characteristics. 

Motor neuron nuclei in trained euploid and trained trisomic mice (Figure 1c,d and Figure 2c,d) showed more irregular shape than their untrained counterparts, and perichromatin granules appeared more numerous; in addition, nucleoli contained larger fibrillar centres in trained trisomic mice (Figure 2d). 

### 3.2. Ultrastructural Morphometry

Data were obtained from sedentary euploid (*n* = 2), sedentary trisomic (*n* = 2), trained euploid (*n* = 2), and trained trisomic (*n* = 2) mice.

**Figure 3:**
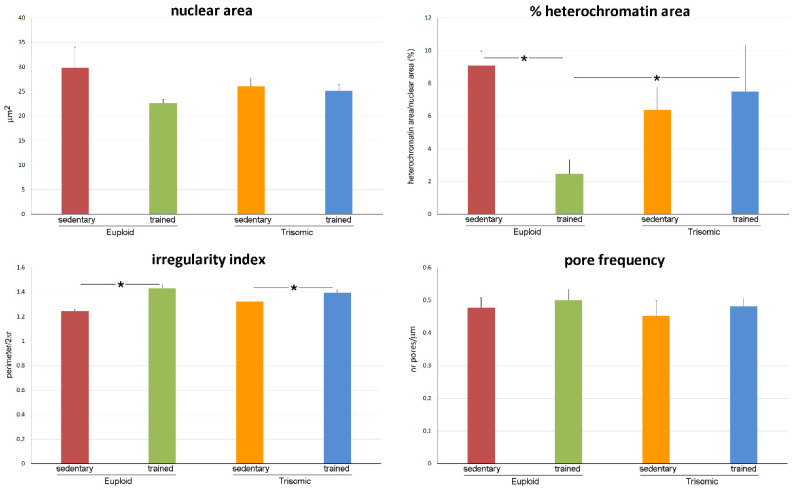
The histograms show the mean values ± SEM of various nuclear variables measured in motor neurons of sedentary euploid, sedentary trisomic, trained euploid, and trained trisomic mice. Asterisks indicate statistically significant difference.

The surface area of motor neuron nuclei did not show any statistically significant difference in the four groups (*p* = 0.729) (Figure 3). The percentage of nuclear area occupied by heterochromatin showed a statistically significant difference in the four groups (*p* = 0.034) (Figure 3); post hoc analysis showed that this variable was not statistically significant different in sedentary euploid vs. sedentary trisomic mice (*p* = 0.487) and decreased in trained euploid vs. sedentary euploid mice (*p* < 0.001). No statistically significant difference was found in sedentary trisomic vs. trained trisomic mice (*p* = 0.729). 

The index of nuclear shape irregularity showed statistically significant difference in the four groups (*p* < 0.001); post hoc analysis showed a statistically significantly increase in both trained euploid and trained trisomic mice vs. their respective sedentary mates (*p* < 0.001 for both); no statistically significant difference was found in sedentary euploid vs. sedentary trisomic mice (*p* = 0.213) (Figure 3). No statistically significant difference was found for nuclear pore frequency in the four groups (*p* = 0.810) (Figure 3).

**Figure 4:**
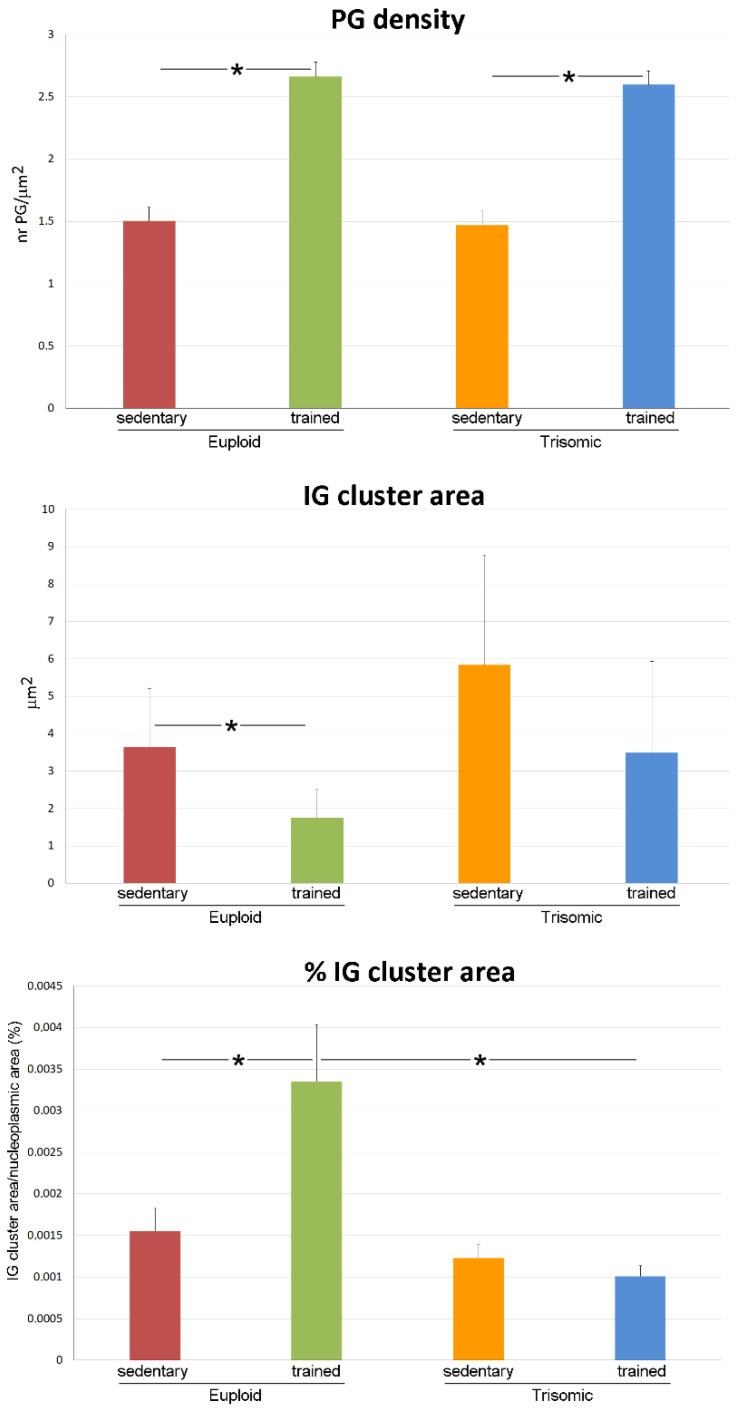
The histograms show the mean values ± SEM of various nucleoplasmic variables measured in motor neurons of sedentary euploid, sedentary trisomic, trained euploid, and trained trisomic mice. PG, perichromatin granules; IG, interchromatin granules. Asterisks indicate statistically significant difference.

In the nucleoplasm, perichromatin granules density showed statistically significant difference in the four groups (*p* < 0.001); post hoc analysis showed no statistically significant difference in sedentary euploid vs. sedentary trisomic mice (*p* = 0.762) and a statistically significantly increase in both trained euploid and trained trisomic vs. sedentary euploid and sedentary trisomic mice, respectively (*p* < 0.001 for both) (Figure 4). 

The size of the interchromatin granules clusters (Figure 4) showed a statistically significant difference in the four groups (*p* = 0.022); post hoc analysis showed no statistically significant difference in sedentary euploid vs. sedentary trisomic mice (*p* = 0.399) and a statistically significantly decrease in trained euploid vs. sedentary euploid mice (*p* = 0.005). No statistically significant difference was found in sedentary trisomic vs. trained trisomic mice (*p* = 0.852). The percentage of nucleoplasmic area occupied by interchromatin granules clusters showed a statistically significant difference in the four groups (*p* < 0.001) (Figure 4); post hoc analysis showed no statistically significant difference in sedentary euploid vs. sedentary trisomic mice (*p* = 0.306) and a statistically significant increase in trained euploid vs. sedentary euploid mice (*p* = 0.014). No statistically significant difference was found in sedentary trisomic vs. trained trisomic mice (*p* = 0.216).

**Figure 5:**
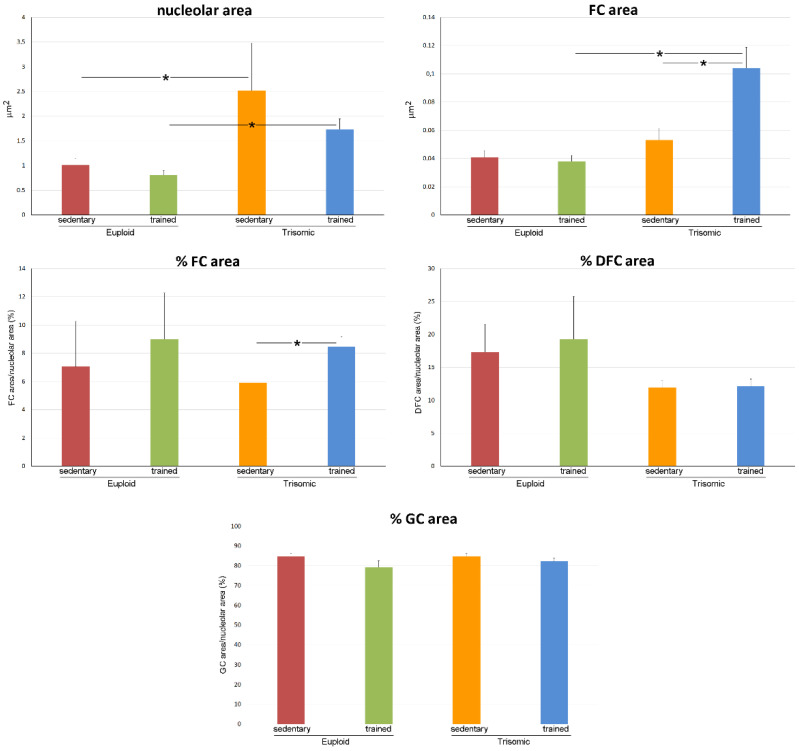
The histograms show the mean values ± SEM of various nucleolar variables measured in motor neurons of sedentary euploid, sedentary trisomic, trained euploid and trained trisomic mice. FC, fibrillar centre; DFC, dense fibrillar component; GC, granular component. Asterisks indicate statistically significant difference.

The nucleolus size showed a statistically significant difference in the four groups (*p* = 0.035); post hoc analysis showed larger nucleolus size in sedentary trisomic vs. sedentary euploid mice (*p* = 0.001) (Figure 5), while no statistically significant difference was found in sedentary euploid vs. trained euploid mice (*p* = 0.607) as well as sedentary trisomic vs. trained trisomic mice (*p* = 0.152).

Morphometric analysis of the nucleolar structural components showed that fibrillar centre area was statistically significant different in the four groups (*p* < 0.001); post hoc analysis showed no statistically significant difference in sedentary euploid vs. sedentary trisomic mice (*p* = 0.581) and a statistically significant increase in trained trisomic vs. sedentary trisomic mice (*p* < 0.001) (Figure 5). The percentage of nucleolar area occupied by fibrillar centre was statistically significant different in the four groups (*p* = 0.041); post hoc analysis showed no statistically significant difference in sedentary euploid vs. sedentary trisomic mice (*p* = 0.171) as well as sedentary euploid vs. trained euploid mice (*p* = 0.086) and a statistically significantly increase in trained trisomic vs. sedentary trisomic mice (*p* = 0.001) (Figure 5). The percentage of nucleolar area occupied by dense fibrillar component and granular component did not show statistically significant difference in the four groups (*p* = 0.303 and *p* = 0.307, respectively) (Figure 5).

### 3.3. Ultrastructural Immunocytochemistry

Data were obtained from sedentary euploid (*n* = 2), sedentary trisomic (*n* = 2), trained euploid (*n* = 2), and trained trisomic (*n* = 2) mice.

**Figure 6:**
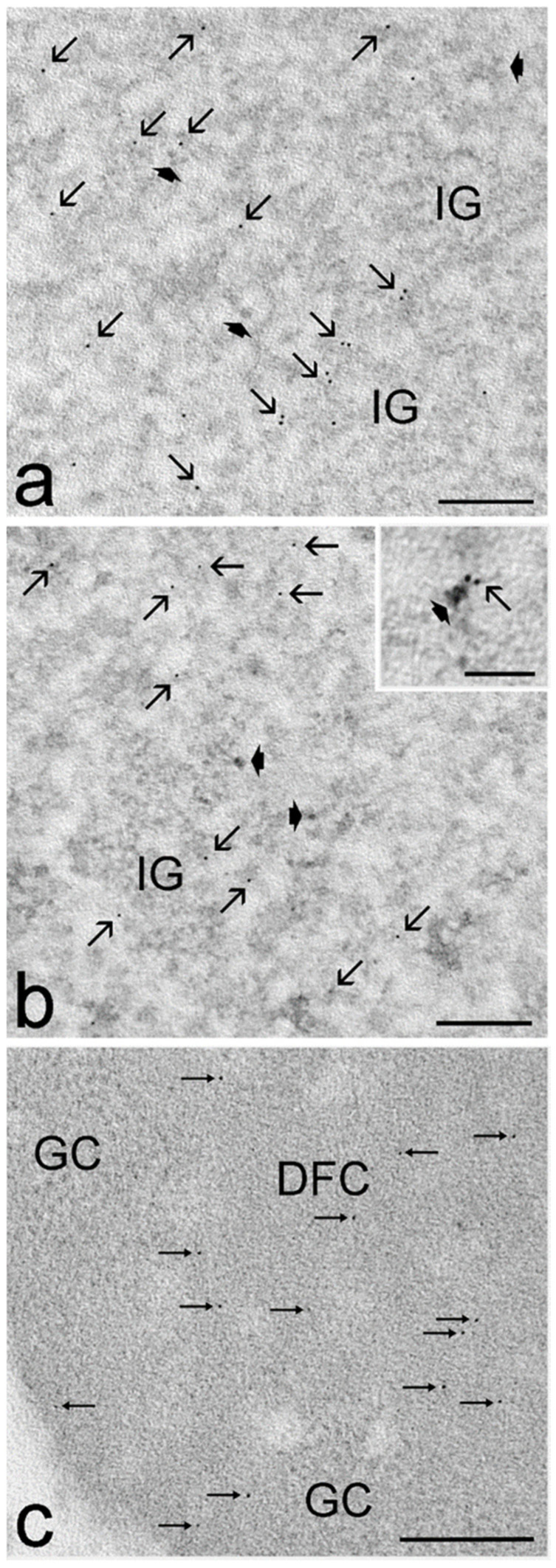
Representative transmission electron micrographs of motor neuron nuclei immunolabelled for polymerase II (**a**), (Sm)snRNP core protein (**b**), and fibrillarin (**c**). (**a**) Trained euploid mouse. Anti-polymerase II antibody specifically labels perichromatin fibrils (arrows), while perichromatine granules (arrowheads) and interchromatin granules are devoid of labelling. (**b**) Sedentary trisomic mouse. Anti-(Sm)snRNP core protein antibody is preferentially distributed on perichromatin fibrils (arrows). The inset show a high magnification image of a labelled perichromatin fibril (arrow) connected to a perichromatin granule (arrowhead). (**c**) Sedentary euploid mouse. Anti-fibrillarin antibody (thin arrows) specifically labels the nucleolar dense fibrillar component but not the granular component. Bars, 200 nm; inset, 100 nm.

The distribution of immunolabelling for the phosphorylated polymerase II, (Sm)snRNP core protein and fibrillarin was similar in motor neuron nuclei of sedentary euploid, sedentary trisomic, trained euploid, and trained trisomic mice; polymerase II (Figure 6a) and (Sm)snRNP core protein (Figure 6b) were almost exclusively associated with perichromatin fibrils, and fibrillarin (Figure 6c) occurred specifically on the nucleolar dense fibrillar component.

**Figure 7:**
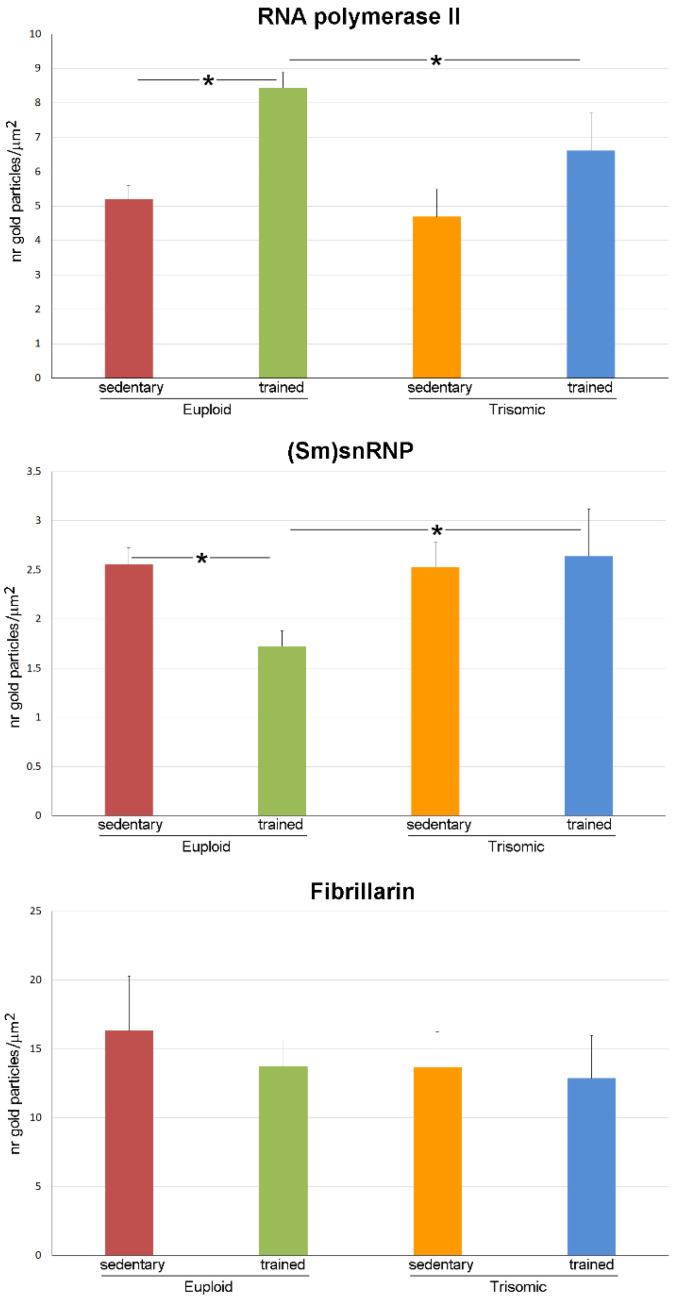
The histograms show the mean values ± SEM of labelling densities of polymerase II, (Sm)snRNP core protein and fibrillarin in motor neuron nuclei of sedentary euploid, sedentary trisomic, trained euploid, and trained trisomic mice. Asterisks indicate statistically significant difference.

Quantitative analysis of the immunolabelling showed a statistically significant difference in the four groups for both polymerase II (*p* < 0.001) and (Sm)snRNP core protein (*p* = 0.042) density. Post hoc analysis revealed a statistically significant increase in polymerase II density in nuclei of trained euploid vs. sedentary euploid mice, sedentary trisomic, and trained trisomic mice (*p* < 0.001 for all). No statistically significant difference was found in trained trisomic vs. sedentary trisomic mice (*p* = 0.057). A statistically significant decrease in (Sm)snRNP core protein density was found in nuclei of trained euploid vs. sedentary euploid, sedentary trisomic, and trained trisomic mice (*p* = 0.004, *p* = 0.017 and *p* = 0.031, respectively). No statistically significant difference was found for fibrillarin labelling density in the four group (*p* = 0.698) (Figure 7). 

Background values were negligible in all the immunolabelling experiments (not shown).

## 4. Discussion

Trisomic Ts65Dn mice have motor dysfunctions similarly to humans with DS. Some investigation has been conducted in CNS regions involved in motor coordination, such as the hippocampus or cerebellum [22,42,43,44,45], but, to the best of our knowledge, ultrastructural studies on motor neurons of the Ts65Dn murine model of DS are lacking. Motor neurons are responsible for the transmission of signals from the CNS to skeletal muscles making direct contact with myofibers trough the neuromuscular junction. Therefore, understanding the effects of trisomy on these nerve cells could shed light on the mechanisms underlying the impaired motor activity typical of DS and the effect of treatment.

The fine architecture of cell nuclei and changes of constituents therein reflects the functional state of the cell [46]. The ultrastructural analysis carried out in this work on the motor neuron nuclei of Ts65Dn mice demonstrated that trisomy per se do not affect most of the nuclear features, such as nuclear area and shape, nuclear pore frequency, heterochromatin, the amount of perichromatin granules or interchromatin granules, or the organization of the nucleolar structural components. The only exception was the nucleolar size, which was statistically significant larger in trisomic mice, maybe due to the spatial rearrangement of the genome caused by the extra Hsa21 [47]. The absence of main structural alterations in nuclear components is consistent with the absence of nuclear functional changes, as shown by immunolabelling for mRNA and rRNA transcription and splicing factors. 

The only study on neuron cell nuclei in Ts65Dn mice was conducted by [30] on hippocampal granular cells. In this work, decrease in nuclear size with increased heterochromatin, nuclear fragmentation and reduced mRNA transcriptional and post-transcriptional activity were found in trisomic mice, suggesting a relationship between such nuclear alterations and the typical DS cognitive dysfunctions. Results of the present study showed that motor neurons are not affected with such nuclear changes, which is consistent with previous observations showing that the trisomy-related dysregulation of gene expression may differently affect organs and tissues [48,49].

In the absence of main nuclear alterations in sedentary trisomic vs. euploid mice, findings presented herein indicate that motor neuron nuclei of trisomic mice are sensitive to the physical training stimulus, although at a lesser extent than euploid. 

In euploid mice, physical training was associated with an increase in nuclear shape irregularity, reduction in heterochromatin, increase in perichromatin granules amount, as well as an increase in total interchromatin granules (redistributed in smaller clusters), increase in polymerase II, and decrease in (Sm)snRNP core protein. All of these changes suggest increased transcriptional and post-transcriptional activity as previously demonstrated in muscle cell nuclei of old mice undergoing adapted physical training [50,51]. The decrease in heterochromatin amount indicates a process of euchromatization that occurs when transcriptional activity increases, thereby requiring higher amounts of the activated form of the transcriptional factor polymerase II. Perichromatin granules form from perichromatin fibrils [52] and contain already spliced pre-mRNA ready to be exported [53]; generally, when mRNA production overcomes its cytoplasmic utilization, the number of perichromatin granules increases [52]. The decrease in (Sm)snRNP core protein, an early splicing factor typically occurring on perichromatin fibrils [39], may be related to the increased transcriptional rate that fasters perichromain fibrils formation [54] and their transformation into perichromatin granules. The overall increase in interchromatin granules, representing the storage, assembly, and phosphorylation sites for transcription and splicing factors [55], may again be related to the increased request of factors for pre-mRNA processing, while their distribution in clusters of smaller size in comparison to the sedentary condition would facilitate the traffic of such factors between interchromatin granules and the interchromatin space where they play their functions. Finally, cell nuclei become more irregular in shape when the cell metabolic rate increases (see, e.g., [56,57]); in fact, a larger nucleus–cytoplasm interface helps the molecular trafficking between these two cellular compartments.

In trisomic mice, physical training only induced an increase in perichromatin granules density and nuclear shape irregularity, with polymerase II showing an increase at the limit of the statistical significance; moreover, an enlargement of fibrillar centres in the nucleoli was observed in trained trisomic mice (but not in trained euploid mice). This suggests that adapted physical training as administered in this study stimulates pre-mRNA transcription and processing activity in motor neuron nuclei of trisomic mice, but at a much lesser extent than in their euploid mates. Interestingly, physical training proved able to also modify the nucleolar architecture of the trisomic mice, impacting the nuclear compartment undergoing specific alteration due to trisomy. In particular, physical training induced an increase in the fibrillar centre component, which contains non-transcribing rDNA [58]. It could be hypothesized that the increase in fibrillar centres is due to a euchromatization of ribosomal genes; however, this was not paralleled by an increase in rRNA transcription, as demonstrated by the steady amount of dense fibrillar component (site of pre-rRNA transcription and splicing, [32]) and fibrillarin therein (pre-RNA early splicing factor, [40]). All motor nuclei changes reported herein are independent of the possible acute effect of exercise because analysis was carried out three days after the last treadmill session; instead, they seem to reflect a kind of chronic adaptation. 

## 5. Conclusions

Our findings showed that adapted physical training chronically stimulates the activity of medullary motor nuclei in trisomic Ts65Dn mice, although to a limited extent vs. euploid mates. The results were obtained from adult animals, i.e., when the consolidation of muscular deficit already occurred [19], but signs of early neuromuscular senescence are not yet ensued [59]. Although based on a limited number of animals due to both poor breeding capability and frailty of Ts65Dn strain, the present findings provide an experimental background to the investigation of the mechanisms underlying the positive effects of physical activity in DS. 

## Data Availability

Data are contained within the article. Additional data are available from the corresponding author, upon reasonable request.
